# Maxillary Hypoplasia and Non-Invasive Ventilation: Literature Review and Proposed New Treatment Protocol

**DOI:** 10.3390/children11060720

**Published:** 2024-06-13

**Authors:** Maria Costanza Meazzini, Mattia Moretti, Gabriele Canzi, Davide Sozzi, Giorgio Novelli, Fabio Mazzoleni

**Affiliations:** 1 Pediatric Craniofacial Malformations Unit, Smile House Monza—Craniofacial Center, Fondazione IRCCS San Gerardo dei Tintori, 20900 Monza, Italy; maria.meazzini@irccs-sangerardo.it (M.C.M.); fabio.mazzoleni@irccs-sangerardo.it (F.M.); 2 Operative Unit of Maxillo-Facial Surgery, Fondazione IRCCS San Gerardo dei Tintori, 20900 Monza, Italy; davide.sozzi@unimib.it (D.S.); giorgio.novelli@unimib.it (G.N.); 3 Postgraduate School of Maxillo-Facial Surgery, University of Milan, 20122 Milan, Italy; 4 Maxillo-Facial Surgery Unit, ASST Grande Ospedale Metropolitano Niguarda, 20162 Milan, Italy; gabriele.canzi@ospedaleniguarda.it; 5 Department of Medicine and Surgery, School of Medicine and Surgery, University of Milan-Bicocca, 20900 Monza, Italy

**Keywords:** non-invasive ventilation, positive airway pressure, midfacial hypoplasia, trans-sutural distraction, Alt-RAMEC

## Abstract

The impact of non-invasive ventilation (NIV) on pediatric maxillary growth is a subject of ongoing research considering its increased use in the pediatric population due to technological advancements and broader indications. This review examines the existing literature, encompassing original articles, case reports, and reviews, to evaluate the effects of NIV on maxillary development and explore potential treatment options. Although the majority of studies agree on the adverse effects of prolonged NIV on maxillary development, techniques for its correction remain understudied. Introducing a novel treatment protocol, we addressed the challenge of correcting severe midfacial hypoplasia in a child with congenital central hypoventilation syndrome (CCHS) undergoing NIV therapy, thus sidestepping the necessity for osteotomies. This proposed protocol holds promise in correcting the adverse impact of NIV on maxillary growth, emphasizing the need for further exploration into innovative treatment modalities.

## 1. Introduction

Non-invasive ventilation (NIV) is defined in *Medical Subject Headings* (MeSH) as a set of techniques for administering artificial respiration without the need for intra-tracheal intubation, predominantly in the form of positive-pressure ventilation/ventilatory assistance [1]. Its use in the pediatric population has increased in recent decades, particularly in cases of obstructive sleep apnea syndrome (OSAS), neuromuscular disorders, congenital central hypoventilation syndrome (CCHS), and cystic fibrosis, to support ventilation through an external airway interface [2,3,4].

The introduction of NIV into clinical practice has significantly improved survival rates and reduced morbidity in pediatric patients, facilitating home management and consequently reducing hospitalization rates and improving the quality of life for these patients [5]. The increase in the associated literature in recent decades reflects the expanded use of NIV thanks to technological advancements and broader indications, especially for pediatric patients.

Additionally, these techniques help mitigate long-term issues associated with invasive ventilation or tracheostomy, both clinically—such as infections, bleeding, dysphagia, and delayed speech development—and managerially, including psychological stress and financial impact on families [6].

However, the effectiveness of NIV, regardless of the underlying pathology, relies on maintaining an airtight seal between the ventilator interface and the facial skin, often achieved using headgear straps [7], to prevent mask displacement and air leaks. The consequent application of constant pressure over long periods, however, appears to have negative effects [8], including skin lesions, claustrophobia, and midface hypoplasia [9]. Maxillary and midface hypoplasia, in fact, seems to be a complication recently identified in the literature, likely due to the continuous compression of circum-maxillary sutures during growth. However, its incidence and treatment are not fully understood. There are still few published studies on this topic, and the case series are extremely limited. For this reason, we decided to conduct a narrative review of the literature on maxillary hypoplasia in pediatric patients undergoing non-invasive ventilation to define the current state of knowledge and propose an innovative treatment protocol.

## 2. Review Section

### 2.1. Database Search

A search was conducted in April 2024 on PubMed and MEDLINE databases using variously combined keywords: “Non-invasive ventilation”, “NIV”, “Positive airway pressure”, “PAP”, “Continuous positive airway pressure”, “CPAP”, “Midface growth”, and “Maxillary hypoplasia”. A search of the references in the included studies was then conducted to identify additional papers. Inclusion and exclusion criteria used in this review are detailed in Table 1. 

Two authors (M.C.M. and M.M.) independently removed duplicate papers and selected the studies for final inclusion. 

### 2.2. Included Studies

The literature regarding the influence of NIV on craniofacial growth is limited: the search identified four case reports or letters to editors, three retrospective studies, one prospective study, and a systematic literature review, as shown in Table 2.

The first case reports framing maxillary hypoplasia as a possible complication of prolonged NIV in growing pediatric patients were published in the early 21st century:

▪ In 2000, Li et al. [10] described a 15-year-old child with OSAS and obesity who underwent CPAP with a nasal mask for 10 years, resulting in midface hypoplasia and a Class III skeletal relationship on lateral cephalometric radiography, despite the absence of known syndromic conditions. However, no description of possible treatment for this dento-skeletal alteration was provided.
▪ In 2002, Villa et al. [11] presented the case of a 7-year-old patient affected by CCHS who had been undergoing bi-level positive airway pressure (BiPAP) since the age of 9 months. The authors attributed the development of maxillary retrusion to prolonged pressure exerted by the ventilator interface and headgear on the zygomatico-maxillary region. In this case, a treatment option involving the application of a Delaire mask over the nasal mask was proposed, leading to normalization of the clinical profile picture.
▪ In 2013, Shibata et al. [13] published another case report highlighting the development of maxillary hypoplasia in a 5-year-old patient with CCHS undergoing BiPAP since the age of 2 months. The authors attributed the midface retrusion observed on lateral cephalometric radiography to growth suppression induced by NIV and described the possibility of mitigating, but not correcting, the clinical picture using a Delaire mask.
▪ In 2017, Haviv et al. [15] reported the case of a 29-year-old patient with myasthenia gravis treated with CPAP since the age of 12 years, exhibiting severe Class III malocclusion, attributed to the use of a tight-fitting nasal mask.

The publication of these case reports prompted the design of three retrospective studies by Fauroux et al. [8] in 2005, Korayem et al. [7] in 2013, and Roberts et al. [14] in 2015, with conflicting results:

▪ In the first study, conducted on 40 children undergoing NIV, global facial flattening was observed in 68% of patients, with maxillary retrusion in 37% of cases, correlated with more than 10 h per day of PAP use.
▪ In the second study, 12 pediatric patients undergoing PAP therapy for at least 6 months and 6 h per day were compared to a control group of 11 patients affected by OSAS not treated with NIV. The authors did not identify associations between midfacial projection and PAP usage in growing patients.
▪ The last study compared 50 children compliant with PAP therapy to 50 non-compliant subjects, analyzing annual cephalometric analyses. The results showed progressive maxillary retrusion in the compliant sample compared to the non-compliant sample.

Tsuda et al. [12] published the first prospective study in 2010 involving 46 children treated with CPAP for at least 2 years and followed with cephalometric tracings. The authors highlighted craniofacial changes with reductions in maxillary and mandibular prominence. 

Finally, Bariani et al. [16] published a systematic literature review in 2020 confirming the association between PAP usage and maxillary hypoplasia.

The majority of the cited articles, except for Korayem et al. [7], agree that prolonged and continuous PAP usage in growing patients has a negative effect on the development of maxilla and midface in the antero-posterior direction. Although the numbers of cases were not extensive, and often the study design did not allow determination of pre-treatment cephalometric status, as presented only by Roberts et al. [14] and Tsuda et al. [12], the possibility of growth constriction induced by pressure applied in the opposite direction to sutural growth seems confirmed. However, only the works of Villa et al. [11] and Shibata et al. [13] propose a possible treatment and demonstrate its efficacy in improving the deformity, albeit partially. 

## 3. Proposal of a New Treatment Protocol

**Figure 1:**
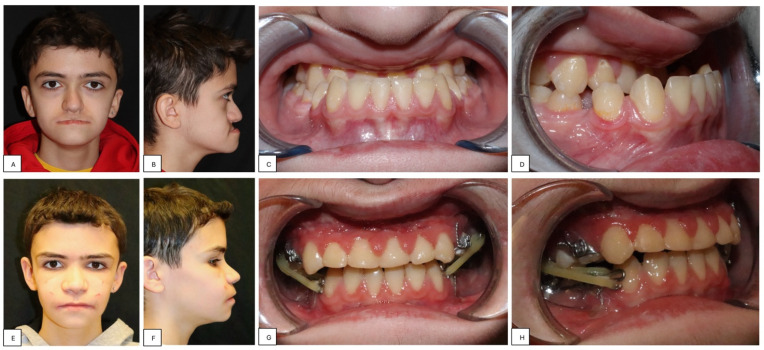
Clinical and occlusal images pre-treatment (**A**–**D**) and post-treatment (**E**–**H**).

**Figure 2:**
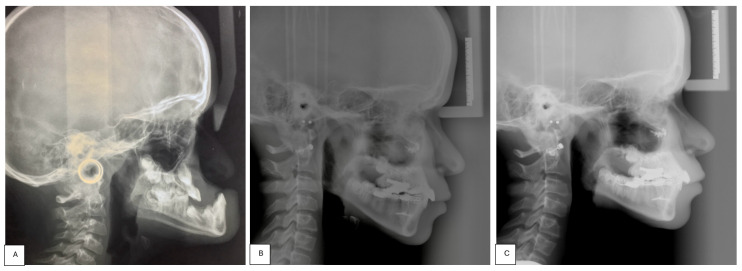
Pre-treatment (**A**), post-treatment (**B**), and 1-year follow-up (**C**) lateral cephalometric radiographs.

A 13-year-old patient affected by CCHS presented to our department with severe retrusion and hypoplasia of the midface, a concave-type facial profile, and a severe skeletal Class III malocclusion (Figure 1 and Figure 2). 

**Figure 3:**
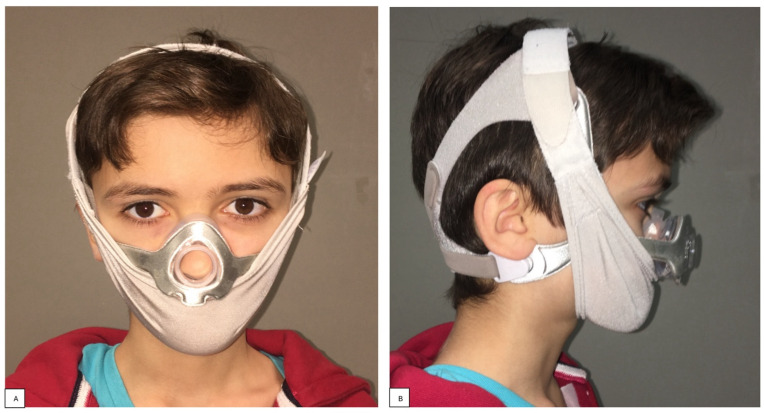
Frontal (**A**) and lateral (**B**) clinical images with NIV interface.

This dysmorphism was partly due to the use of NIV during the night (Figure 3).

The proposed treatment aimed at both aesthetic and functional improvement, as excessive retrusion of the maxilla would lead to loss of lip competence and increased complexity in the adherence of NIV interfaces, potentially causing nocturnal ventilatory issues. 

**Figure 4:**
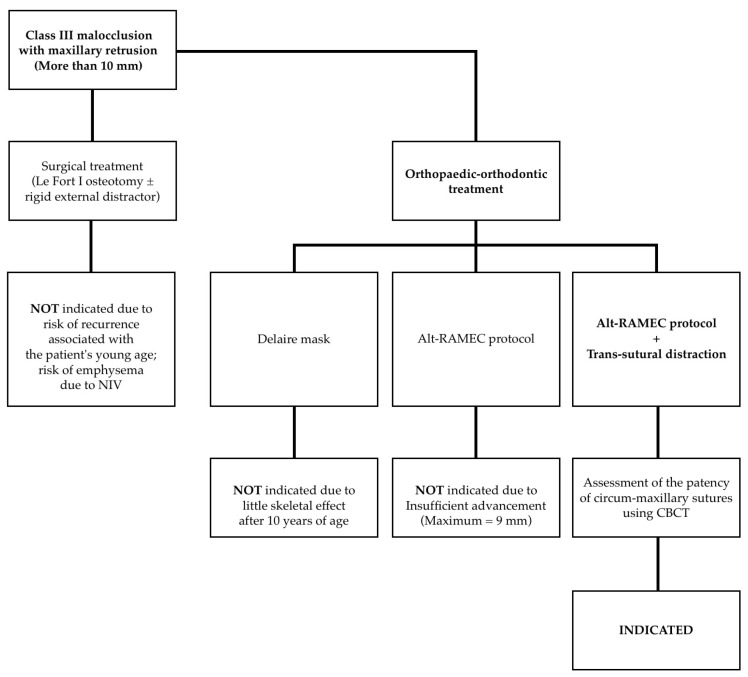
Treatment options.

The various treatment options considered are illustrated in Figure 4.

**Figure 5:**
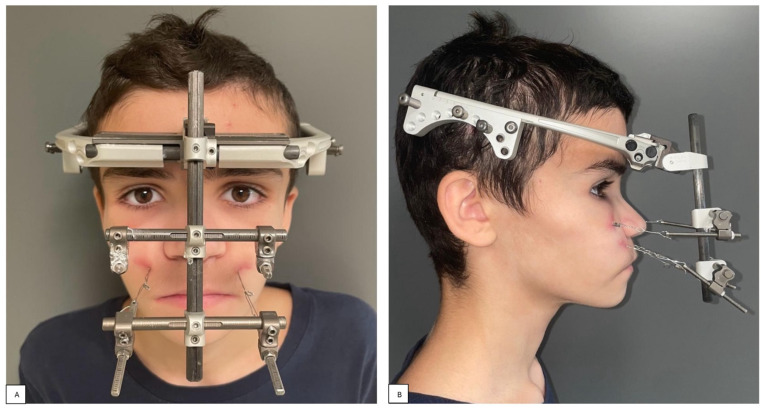
Frontal (**A**) and lateral (**B**) clinical images with rigid external distractor.

Following multidisciplinary discussion and ruling out purely surgical options, which could create communications between the maxillary sinuses and soft tissues, posing a risk of massive facial emphysema secondary to ventilation, the following treatment plan was confirmed in agreement with the patient’s parents:

1. Perform a CBCT to confirm the patency of the circum-maxillary sutures.
2. Placement of a two-hinged rapid maxillary expander (RPE) and expansion of maxilla using a modified Alt-RAMEC (alternate rapid maxillary expansion and constriction) protocol [17,18,19] resulting in loosening of all circum-maxillary sutures without surgery. The treatment protocol consisted of 11 cycles with 7 days of expansion and 7 days of constriction, 1 mm per day, alternatively. After 11 weeks of alternate expansion–constriction, mild mobility of the whole maxilla was felt clinically.
3. Placement of an external rigid distractor (RED) during a minor surgery under general anesthesia. The surgical procedure was performed with oro-tracheal intubation through an incision at the level of the upper vestibular fornix with skeletonization of the maxilla. The patency of the zygomatico-maxillary suture was noted. A plate and corresponding screws (Matrix Midface, DePuy Synthes, Raynham, MA, USA) were positioned on each side at the level of the maxillo-malar complex, and two plates, corresponding screws, and percutaneous pins (External Midface Distractor, DePuy Synthes, Raynham, MA, USA) were placed at the level of the lower orbital rim. Percutaneous traction wires were secured to the inferior plates and left protruding at the level of the naso-labial fold. After all intraoral wounds were sutured, the halo was positioned using four cranial screws per side (External Midface Distractor, DePuy Synthes, Raynham, MA, USA). The immediate post-operative period was organized in the Pediatric Intensive Care Unit. As ongoing NIV therapy was critical, the tractions of the RED were positioned in such a way as not to impede the placement of the NIV interface during night (Figure 5).
4. Activation of the RED to allow advancement of the midface to overcorrect the dysmorphism. The activation started the day after surgery, 0.5 mm per day. After 4 weeks, the dislodgement of a bone anchorage plate was observed, necessitating an additional intervention to reposition it with a modification of its geometry. The activation continued for 5 additional weeks, for a total of 9 weeks, with an advancement reaching approximately 20 mm at the maxillary level.
5. At the end of the distraction phase, after an additional stabilization period of 4 weeks, the RED was removed.
6. Continuation of orthopedic–orthodontic treatment for approximately 9 months using intra-oral traction elastics, following the placement of orthodontic anchorage screws in the jaws under local anesthesia.

## 4. Discussion

Congenital central hypoventilation syndrome (CCHS), also known as Ondine’s curse or syndrome, is a neurological disorder related to mutations in the PHOX2B gene, characterized by the failure of automatic ventilation control during sleep in the absence of primary neuro-muscular, cardio-circulatory, pulmonary, or metabolic alterations.

Patients with this condition require mechanically assisted ventilation for their entire lives. In some cases, tracheostomy may be necessary, but generally, non-invasive positive pressure ventilation (NPPV) or bi-level positive airway pressure (BiPAP) provide, through a mask, adequate support during sleep [20].

The early and prolonged use of positive pressure ventilation devices during growth appears, according to current knowledge, to be a possible cause of maxillary hypoplasia. Indeed, the pressure exerted by the interface as well as the need to keep the facial mask adhered to the face could cause an alteration in the sagittal growth of the upper jaw due to the prolonged compression of the circum-maxillary sutures [16].

Despite the rarity of the condition and the resulting difficulty in standardizing any corrective treatment, the literature has proposed the use of orthodontic appliances and/or facial masks to improve midfacial growth, but with insufficient results [13].

By the end of the 21st century, after some pilot studies on animal models [21,22,23], a technique called trans-sutural distraction was introduced in the literature [24]. This technique allows for skeletal growth induction by applying traction forces at the level of facial sutures without the need for osteotomies. It has been applied with variable results, especially in patients with maxillary hypoplasia resulting from cleft lip and palate [24,25,26].

On the other hand, the introduction into clinical practice of the Alt-RAMEC (alternate rapid maxillary expansion and constriction) protocol proposed by Liou [17] (and its modifications) ensured the possibility of obtaining, through the release of circum-maxillary sutures and subsequent traction, a stable non-surgical advancement of the maxilla of approximately 6 mm in both patients with cleft lip and palate and patients with Class III malocclusion [17,18,19].

The correction of severe maxillary hypoplasia in growing patients is complex and often leads to outcomes characterized by poor stability, especially in patients undergoing non-invasive ventilation, where the compression of the circum-maxillary sutures is constant over time [16].

To the best of our knowledge, this is the first report proposing the use of the Alt-RAMEC protocol followed by trans-sutural distraction with the aid of a rigid external distractor to maximize the possibility of maxillary advancement by loosening the circum-maxillary sutures. Lateral standardized cephalometric X-rays were conducted both before and after treatment. Delta Dent software version 2.2.1 (Outside Format, Pandino, Italy) was utilized to trace all cephalograms. A set of reproducible landmarks were defined and digitized for the performed tracings. Accurate identification of anatomical landmarks facilitated superimposition using a best-fit method on the sella–nasion (SN) plane, aligning pre- and post-treatment radiographs.

In our case, superimposed pre- and post-treatment cephalometric tracings highlighted a marked and stable advancement of the maxilla with a reduction in facial concavity: in particular, the SNA angle improved from 83.6° to 102.5° (Δ = 18.9°), the SNB angle varied from 103.2° to 97.5° (Δ = −5.7°), the ANB angle improved from −19.5° to 5.0° (Δ = 24.5°), as did the SN^GoMe angle, which increased from −10.6° to 18.1° (Δ = 28.7°).

**Figure 6:**
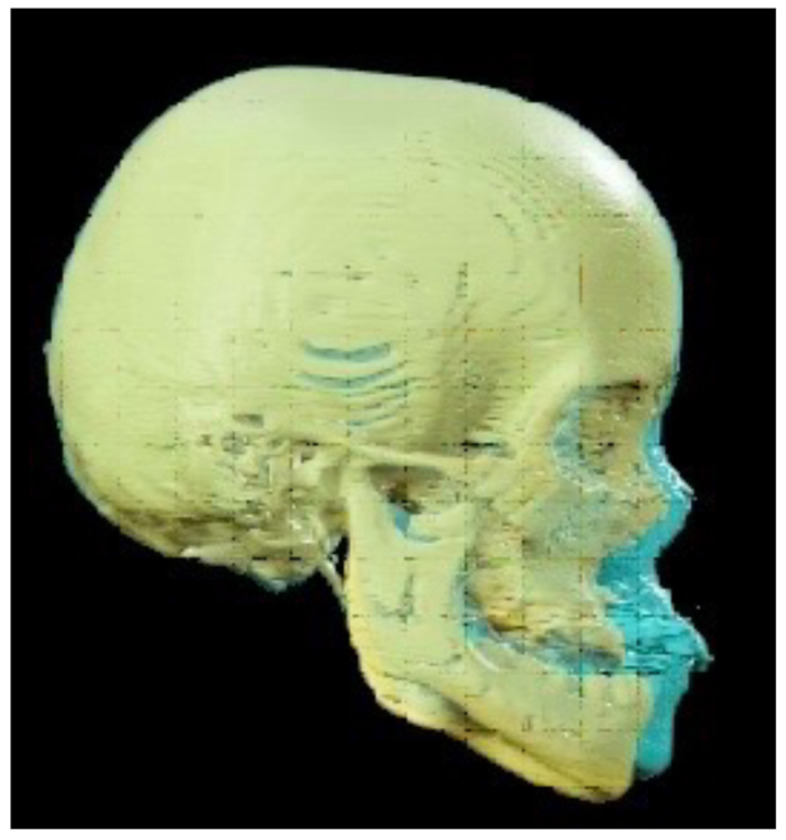
Superimposition of 3D models obtained from CT scans: the skull reconstruction before treatment is highlighted in yellow, while the one after treatment is depicted in blue.

Further interesting results were obtained by analyzing the pre- and post-treatment CT scans and the superimposition of 3D models obtained from their processing. A multislice CT scan of the maxillofacial region was performed before and after treatment. CT data, saved in Digital Imaging and Communications in Medicine (DICOM) format, were imported into BrainLab Elements software version 3.0.6.14 (BrainLab, Munich, Germany) for analysis and superimposition, achieved using autofusion, as visible in Figure 6.

A substantial stability of cranial vault and cranial base positions is highlighted, with a marked advancement of the midface: the advancement was 4.5 mm for the nasion, 14.7 mm for the lower orbital point, and 19.5 mm for the A point. This advancement resembles the osteotomic trajectory of a Le Fort III procedure and exhibits a cranio-caudal gradient with the maximum result achieved at the maxillary alveolar level.

At the level of circum-maxillary sutures, a tensile deformation with bone apposition was observed. This process involved the zygomatico-maxillary, naso-maxillary, and pterygo-maxillary sutures, but also encompassed the zygomatico-frontal, zygomatico-temporal, and naso-frontal sutures. These data are consistent with findings already present in the literature [25,26].

An additional noteworthy finding is the absence of enophthalmos, despite the orbital volume increase due to the advancement of the midface, along with the simultaneous appearance of the typical prominence of the zygoma and the surprisingly aesthetic harmony of the outcome, which might be explained by the gradual distraction of sutures in the absence of osteotomies.

Although the constriction of maxillary growth in children undergoing NIV appears to be well established in the literature, and only Korayem et al. [7] did not find an association between NIV and midface hypoplasia, some of these 12 patients had only 12 months of PAP usage. However, the techniques for its correction remain understudied. Indeed, the effectiveness of treatment with the Delaire mask associated with the ventilation interface seems to be partial and poorly documented. The work of Villa et al. [11] lacks lateral cephalometric radiographs and long-term follow-up, while the conclusions of Shibata et al. [13], supported by radiographic images, indicate the possibility of only partially improving the dysmorphism resulting from NIV. Moreover, these results were obtained in patients younger than 8 years, in whom the Delaire mask treatment has a skeletal effect, which was not possible in our case. Kim et al. [27] have already demonstrated that such treatment is practically ineffective after 12 years of age. Conversely, the Alt-RAMEC protocol can be successfully applied to older patients, who are closer to the end of craniofacial growth (second and third stages of vertebral maturation) [19], but it cannot achieve alone an advancement greater than 9 mm, which was insufficient to correct the dysmorphism in the patient presented in our article. The proposed treatment protocol, on the other hand, facilitated overcoming this limitation, achieving nearly 20 mm of maxillary advancement with the aid of a rigid external distractor. Consequently, our study highlights the possibility of completely correcting severe midfacial hypoplasia without resorting to osteotomies, thus avoiding associated surgical risks. A limitation of the described protocol is the necessity for patent circum-maxillary sutures; for this reason, proper follow-up during growth is required, so as not to miss adequate timing.

## 5. Conclusions

In the current literature, most studies seem to agree on attributing a negative effect on maxillary growth to NIV. In our experience, the presented protocol was effective and applicable in the correction of midfacial hypoplasia, and its results are extremely promising.

Our work also identified the need for further prospective studies, including serial cephalometric analyses over time, of patients undergoing NIV, to precisely define its effects on craniofacial growth and to evaluate the effectiveness of various treatment options.

Further research is necessary to assess the long-term stability of results obtained through the proposed therapeutic protocol, as well as its applicability in forms of maxillary hypoplasia not only of iatrogenic origin, but also associated with craniofacial malformations, such as cleft lip and palate, and craniofacial synostosis.

## Figures and Tables

**Table 1:** Inclusion and exclusion criteria.

Inclusion Criteria	Exclusion Criteria
Human clinical studies	Patients with midface hypoplasia due to other known causes


Publication from any date to April 2024	Full text not in English
Letters to editors, case reports, case series, opinion articles, descriptive studies, retrospective studies, prospective studies, review articles	
Evaluation of the effects of NIV on maxillary growth in pediatric population	

**Table 2:** Studies included.

Authors		Year	Type of Study	Findings
Li et al.	[10]	2000	Case Report	Description of a 15-year-old patient affected by OSAS with midface hypoplasia treated with CPAP.
Villa et al.	[11]	2002	Case Report	Description of a 7-year-old patient affected by CCHS with midface hypoplasia treated with BiPAP. Proposal of a successful treatment option for maxillary hypoplasia involving Delaire mask.
Fauroux et al.	[8]	2005	Retrospective Study	Clinical evaluation of facial side effects in 40 patients affected by OSAS, neuromuscular disorders, and cystic fibrosis treated with non-invasive positive pressure ventilation (NPPV). Maxillary retrusion was present in 37% of patients and was associated with daily use (>10 h per day).
Tsuda et al.	[12]	2010	Prospective Study	Serial cephalometric evaluation of 46 patients affected by OSAS treated with CPAP for a minimum of 2 years. The use of CPAP may cause a significant retrusion of anterior maxilla.
Korayem et al.	[7]	2013	Retrospective Study	Cephalometric evaluation of craniofacial morphology in 12 patients affected by OSAS treated with positive airway pressure (PAP) versus 11 patients affected by OSAS who were not treated with PAP. No association was demonstrated between midface projection and PAP usage in growing patients.
Shibata et al.	[13]	2013	Case Report	Description of a 5-year-old patient affected by CCHS with midface hypoplasia treated with BiPAP. Proposal of a partially successful treatment option for maxillary hypoplasia involving a Delaire mask.
Roberts et al.	[14]	2015	Retrospective Study	Serial cephalometric evaluation in 50 children affected by OSAS compliant with PAP therapy and 50 children affected by OSAS non-compliant with PAP therapy. Midface retrusion was highlighted in patients using PAP for more than 20 h per week for more than 6 months.
Haviv et al.	[15]	2017	Case Report	Description of a 29-year-old patient affected by myasthenia gravis with midface hypoplasia treated with CPAP.
Bariani et al.	[16]	2020	Systematic Review	Analysis of five studies previously published. Midface hypoplasia is associated with long-term use of PAP during growth.

## Data Availability

The original contributions presented in the study are included in the article, further inquiries can be directed to the corresponding author.
